# Validity of self-reported leisure-time sedentary behavior in adolescents

**DOI:** 10.1186/1477-5751-10-2

**Published:** 2011-02-11

**Authors:** Olivia Affuso, June Stevens, Diane Catellier, Robert G McMurray, Dianne S Ward, Leslie Lytle, Melinda S Sothern, Deborah R Young

**Affiliations:** 1 Department of Epidemiology, University of Alabama at Birmingham, 1530 Third Ave, South, RPHB 220E, Birmingham, AL 35294-0022, USA; 2 Department of Nutrition, University of North Carolina at Chapel Hill 245 Rosenau Hall, CB#7461, Chapel Hill, NC 27599-7461, USA; 3 Department of Biostatistics, University of North Carolina at Chapel Hill 137 E. Franklin Street, Suite 203, CB#8030, Chapel Hill, NC 27599-8030, USA; 4 Department of Nutrition, University of North Carolina at Chapel Hill, 305 Wollen Gym, CB#8605, Chapel Hill, NC 27599-8605, USA; 5 Department of Nutrition, University of North Carolina at Chapel Hill, 2206 McGavran-Greenberg, CB#7461, Chapel Hill, NC 27599-7461, USA; 6 Division of Epidemiology and Community Health, University of Minnesota, 1300 S. Second Street, Suite 300, Minneapolis, MN 55454-1015, USA; 7 Division of Behavioral and Community Health Sciences, Louisiana State University, 1615 Poydras Street, Suite 1400, New Orleans, LA 70112-1272, USA; 8 Department of Epidemiology and Biostatistics, University of Maryland, 1242A School of Public Health Building, College Park, MD 20742-0001, USA

## Abstract

**Background:**

To evaluate the concordance between leisure-time sedentary behavior in adolescents assessed by an activity-based questionnaire and accelerometry.

A convenience sample of 128 girls and 73 boys, 11-15 years of age (12.6 ± 1.1 years) from six states across the United States examined as part of the feasibility studies for the Trial of Activity in Adolescent Girls (TAAG). Three days of self-reported time spent watching TV/videos, using computers, playing video/computer games, and talking on the phone was assessed using a modified version of the Self-Administered Physical Activity Checklist (SAPAC). Criterion measure of sedentary behavior was via accelerometry over three days using a cut point of < 50 counts · 30 sec^-1 ^epoch. Comparisons between sedentary behavior by the two instruments were made.

**Results:**

Adolescents generally underestimated minutes of sedentary behavior compared to accelerometry-measured minutes. The overall correlation between minutes of sedentary behavior by self-report and accelerometry was weak (Spearman r = 0.14; 95% CI 0.05, 0.23). Adjustment of sedentary minutes of behavior for total minutes assessed using either percentages or the residuals method tended to increase correlations slightly. However, regression analyses showed no significant association between self-reported sedentary behavior and minutes of sedentary behavior captured via accelerometry.

**Discussion:**

These findings suggest that the modified 3-day Self-Administered Physical Activity Checklist is not a reliable method for assessing sedentary behavior. It is recommended that until validation studies for self-report instruments of sedentary behavior demonstrate validity, objective measures should be used.

## Background

Although a sedentary lifestyle has been identified as a risk factor for adolescent obesity, validated methods to assess sedentary behavior (physical inactivity) are limited due in part to portable criterion methods being developed only recently to measure this construct [1]. Recent studies have examined the use of accelerometry to assess sedentary behavior in controlled conditions and provided population specific accelerometry cut points to indicate a valid measure of sedentary behavior in children [2,3]. Nevertheless, self-report tools remain the most widely used method for assessing behavior in adolescents [4]. In contrast to accelerometry, self-report questionnaires provide a low cost and easy to use method for measuring sedentary behaviors. Questionnaires also have the advantage of capturing the type (e.g. TV viewing) and context (e.g. at home) of sedentary behaviors which may identify key targets for designing efficacious interventions aimed at reducing inactivity.

One of the limitations of self-report behavioral questionnaires is response bias where respondents may intentionally provide incorrect answers to a survey due to pressures to respond in a socially acceptable manner [5-7]. Social desirability, a type of response bias, has been associated misreporting of activity behaviors in both boys and girls [7,8]. Klesges et al. (2004) found that the overestimation of self-reported physical activity was positively associated with social desirability among 8 to 10 year old African American girls. Among 10 to 14 year old boys, social desirability was negatively associated with self-reported sedentary behavior (r = -0.158; p < 0.001). There is some evidence from studies of adults that weight status may also affect reporting of sedentary behaviors, with overweight adults underreporting minutes of sedentary activities compared to normal weight adults [9]. However, the association between weight status and self-reported sedentary behavior has not been examined in youth. In addition, reporting of activity behaviors has been shown to differ by sex in adults [10]. We hypothesized that weight status and sex would influence reporting of sedentary behaviors among adolescents trying to avoid social criticism in a similar manner to that of adults, and therefore affect the validity of self-reported sedentary measures.

Investigators have used questionnaires, such as the Self-administered Physical Activity Checklist (SAPAC) [11], to assess sedentary behaviors [12-14], however, only recently have efforts been made to determine the validity of the self-report measures in free-living participants [15]. The purpose of this research was to evaluate the validity of a three-day self-report physical activity checklist (a modified version of the SAPAC) to assess leisure-time sedentary behaviors in a sample of free-living adolescents using accelerometry as the criterion measure. Overall validity and differences by weight status and sex were examined. We also compared self-reported minutes of sedentary behavior to accelerometry-measured sedentary behavior using three different expressions: 1) unadjusted sedentary minutes, 2) percentage of sedentary minutes, and 3) residuals of predicted sedentary minutes. The inclusion of comparisons of the three methods for estimating concordance was used to explore the effects of adjusting the minutes of sedentary behavior as a function of total time assessed and the within-person variation in sedentary behavior. The aforementioned analytic strategies are common practice in validation studies of self-reported dietary intake [16]. To our knowledge, this study is the first to examine validity of reported leisure-time sedentary behaviors from the SAPAC among adolescent girls and boys.

## Results

### Sample Characteristics

Characteristics for the study sample and the 3-day sedentary behavior assessments are presented in Table 1. The sample (N = 201) included a wide range of body sizes, with 36% of the sample overweight (BMI ≥ 85th percentile on the CDC growth charts). The sample was ethnically diverse: 40% of the sample was minority students and included 15% African American, 12% Multiracial, 9% Hispanic, 3% Asian, and 2% American Indian. Girls spent twice as much time talking on the phone as boys, while boys spent approximately three times the number of minutes playing computer/video games as girls. There were no significant differences by sex for time spent watching TV/videos or using computers/internet. There was also no significant difference in the 3-day average accelerometer-measured minutes of sedentary behavior when stratified by sex.

**Table 1:** Mean (95% CI) characteristics of the sample of 201 adolescents

		Girls		Boys		Combined
						
	N	mean (95% CI), %	N	mean (95% CI), %	N	mean (95% CI), %
Age (years)	128	12.6 (12.4, 12.8)	73	12.6 (12.4, 12.9)	201	12.6 (12.4, 12.7)
Height (cm)	128	157.5 (156.0, 158.9)	73	158.2 (155.6, 160.8)	201	157.7 (156.4, 159.1)
Weight (kg)	128	55.9 (53.5, 58.5)	73	53.7 (49.4, 58.2)	201	55.2 (52.9, 57.4)
BMI category (%)						
< 85th percentile		59		73		64
≥ 85th percentile		41		27		36
Ethnicity (%)						
African American		14		15		15
American Indian		1		4		2
Asian		2		3		3
Multiracial		14		9		12
Hispanic		8		12		9
White		61		57		60
Accelerometer Sedentary Behaviors (mins)	122	354.6 (342.1, 365.8)	68	338.5 (318.8, 358.2)	190	349.3 (339.1, 359.4)
Self-reported Sedentary Behaviors† (mins)						
TV/Video watching	122	174.3 (148.5, 200.1)	68	152.4 (119.9, 184.9)	190	166.1(146.2, 186.4)
Computer/Internet	122	62.2 (43.1, 81.3)	68	39.7 (20.7, 58.7)	190	54.0 (40.1, 67.9)
Talking on phone	122	71.3 (51.2, 92.1)*	68	36.5 (10.5, 62.6)*	190	58.9 (42.7, 74.9)
Video/Computer games	122	15.8 (8.2, 23.4)*	68	43.6 (24.5, 62.6)*	190	25.9 (17.3, 34.4)

* Difference in mean minutes by sex;†Sedentary behavior from modified SAPAC

Overweight girls tended to report fewer minutes of sedentary behavior than normal weight girls, but this observation was not supported by accelerometry data. The accelerometry measures indicated that overweight girls significantly under-reported minutes of sedentary behavior (260 mins. vs. 365 mins.; p = 0.0009). In boys, reported and accelerometry-measured sedentary behavior was similar across weight status groups. However, normal weight boys reported significantly fewer minutes of sedentary behavior compared to accelerometry (264 mins. vs. 334 mins.; p = 0.0161).

Comparisons within groups by sex showed that for individual sedentary behaviors from the modified SAPAC, overweight girls reported fewer mean minutes of TV/video watching (143.8 mins. vs. 191.6 mins.), computer/internet use (50.0 mins. vs. 66.4 mins.), video/computer game playing (14.2 mins vs. 16.7 mins.), and talking on the phone (67.6 mins. vs. 69.9 mins.) compared to normal weight girls. Overweight boys reported more minutes of computer/internet use (40.9 mins vs 39.2 mins.), video/computer game playing (63.1 mins vs. 34.8 mins.), and talking on phone (40.7 mins vs. 34.2 mins.), but not TV/video watching (129.0 mins vs. 155.2 mins.) compared to normal weight boys.

Minutes of TV/video watching as assessed by self-report were significantly correlated with objectively measured sedentary minutes in normal weight and overweight girls (r = 0.21, 95% CI 0.07, 0.35, r = 0.28; 95% CI 0.11, 0.43, respectively). No significant correlations between objectively measured sedentary minutes and self-reported TV/video watching were found in boys. Neither self-reported video/computer games nor talking on the phone were correlated with accelerometry in girls or boys. In contrast, self-reported minutes of computer/internet use were modestly correlated with objectively measured sedentary minutes in normal weight boys (r = 0.26, 95% CI 0.07, 0.43), but not in girls or overweight boys.

Both Spearman and Pearson correlations between self-report and accelerometry by method of analysis are presented in Table 2. The overall 3-day Spearman correlation between self-reported and accelerometry-measured minutes of sedentary behavior for all subjects combined was weak (r = 0.14; 95% CI, 0.05, 0.23). When stratified by sex, Spearman correlations tended to be slightly higher in girls (r = 0.16; 0.05, 0.27) than in boys (r = 0.11; -0.05, 0.26). There were no significant differences in these correlations by sex or weight status. When the minutes of sedentary behavior were adjusted for total minutes of activity assessed by either the percentage or residuals method, the adjusted correlation coefficients tended to increase agreement from the unadjusted estimates. However, the residuals method tended to produce the most precise estimates as evident by smaller confidence intervals. Although in some instances the Pearson correlation coefficients were higher than the Spearman coefficients, none were significantly different as evidenced by the overlapping confidence intervals.

**Table 2:** Spearman and Pearson correlation coefficients for comparison of self-report* and accelerometer minutes of sedentary behavior, both unadjusted and adjusted for total minutes of activity

	Unadjusted		Percentages**		Residuals***	
Spearman correlations	95% CI		95% CI		95% CI	
All Participants	0.14	0.05, 0.23	0.21	0.12, 0.30	0.19	0.10, 0.28
Girls	0.16	0.05, 0.27	0.21	0.10, 0.31	0.21	0.10, 0.31
Boys	0.11	-0.05, 0.26	0.25	0.10, 0.40	0.27	0.11, 0.40
Normal weight	0.20	0.09, 0.30	0.27	0.16, 0.37	0.27	0.16, 0.37
Girls	0.22	0.07, 0.35	0.25	0.11, 0.38	0.23	0.13, 0.33
Boys	0.24	0.04, 0.41	0.32	0.13, 0.48	0.34	0.16, 0.50
Overweight	0.08	-0.07, 0.24	0.16	0.00, 0.31	0.07	-0.08, 0.21
Girls	0.20	0.03, 0.36	0.23	0.06, 0.40	0.20	0.07, 0.31
Boys	-0.29	-0.56, 0.03	0.16	-0.16, 0.45	0.24	-0.08, 0.51
Pearson correlations						
All Participants	0.18	0.07,0.28	0.23	0.12, 0.33	0.16	0.05, 0.27
Girls	0.07	-0.09,0.22	0.30	0.15, 0.43	0.24	0.08, 0.38
Boys	0.13	0.05, 0.22	0.21	0.12, 0.29	0.14	0.05, 0.22
Normal weight	0.03	-0.08, 0.14	0.25	0.11, 0.38	0.17	0.06, 0.28
Girls	0.17	0.03, 0.31	0.33	0.14, 0.48	0.19	0.09, 0.29
Boys	-0.21	-0.37, -0.04	0.24	0.13, 0.34	0.26	0.07, 0.43
Overweight	-0.06	-0.21, 0.05	0.27	0.11, 0.43	0.12	-0.02, 0.26
Girls	0.03	-0.16, 0.22	0.23	-0.08, 0.50	0.24	0.13, 0.36
Boys	-0.37	-0.61, -0.08	0.21	0.07, 0.34	0.30	-0.01, 0.55

* Self-reported sedentary behavior from the modified SAPAC; ** Sedentary minutes divided by total minutes; ***Residuals from regression of total minutes assessed on sedentary minutes

**Figure 1:**
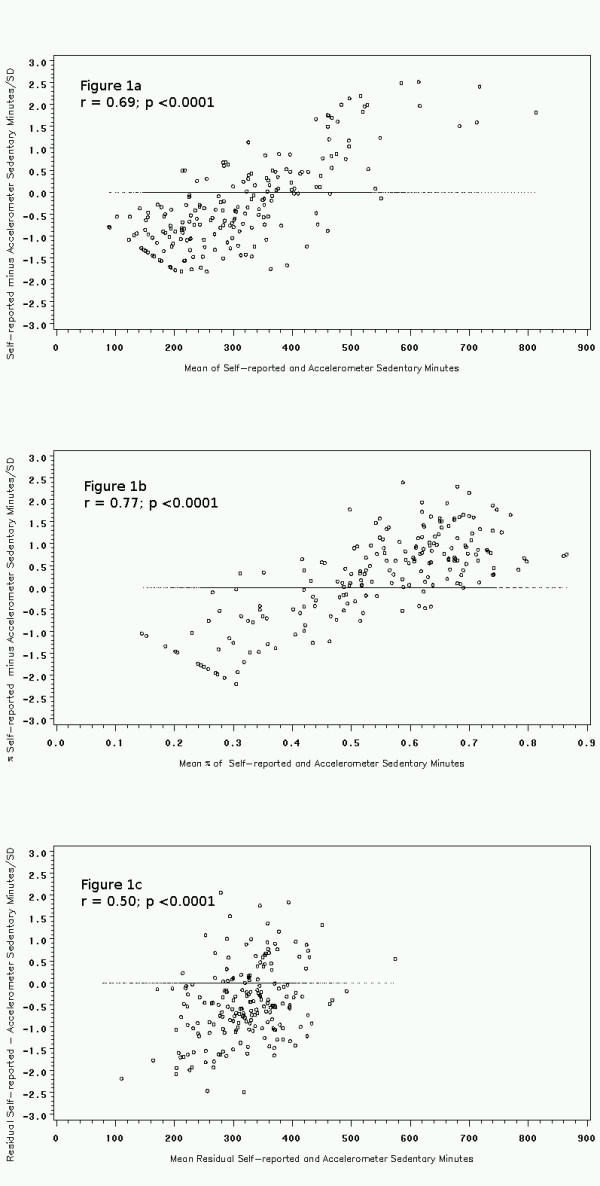
**Bland-Altman plots of sedentary behavior from self-report versus accelerometry standardized to 1 SD**. The results represent the 3 different methods used: 1a) unadjusted minutes, 1b) percent of minutes, and 1c) residual minutes. Mean error scores are shown in each plot.

Bland-Altman plots were used to examine differences between self-report and accelerometry across mean minutes of sedentary behavior by each of the analysis methods (Figures 1a-c.). The scale of the Bland-Altman plots was standardized to allow comparisons between these methods. For unadjusted estimates (Figure 1a.), adolescents under-reported sedentary behaviors at low levels of mean sedentary behavior with under-reporting decreasing as sedentary minutes increased. When adjusted for total daily activity (Figure 1b.), there was less absolute agreement between the self-report and accelerometry sedentary behavior with less under-reporting at low levels of sedentary and increasing over-reporting a higher measures of sedentary behavior. Finally, the correction for within-person variation using the residuals (Figure 1c.) from a regression of sedentary behavior given total activity seemed to produce the smallest absolute difference between self-report and accelerometry across the average minutes of sedentary behavior. Under-reporting decreased as minutes of sedentary behavior increased. This method also produced the most precise measures of comparability between the instruments. For all adolescents combined, overall sedentary behavior below an average of 400 minutes was underestimated by self-report compared to the accelerometer. When stratified by sex and weight status, this pattern remains consistent across plots (data not shown).

In the full regression model in which self-reported sedentary behavior was the dependent variable, accelerometer-measured sedentary behavior was the independent variable, and day, age, grade, sex, ethnicity, and weight status were included as covariates, only day of assessment was significant, F(3,271) = 6.68, p = 0.0002. However, in the reduced model, neither day nor the interaction of day and accelerometer-measured sedentary behavior were significantly related to self-reported sedentary behavior (day, F(3, 272) = 1.15, P = 0.3309; accelerometer *day, F(3,272) = 0.49, p = 0.6891).

## Discussion

The overall Spearman rank-order correlation between self-reported minutes of sedentary behaviors from the modified 3-day SAPAC and accelerometer-measured minutes of sedentary behavior was weak indicating that the questionnaire had inadequate ability to rank students according to their minutes of sedentary behavior. The Spearman correlation tended to increase slightly after adjusting the minutes of sedentary behavior by total minutes assessed using either percentages or the residuals method. In some cases, the Pearson correlation coefficients were greater than the Spearman correlation. However, there were not significant differences between the two methods. Finally, the repeated measures regression analyses showed no association between the self-reported and accelerometer-measured sedentary behaviors after controlling for age, ethnicity, day of assessment, sex, and weight status.

To our knowledge, this study is the first attempt to validate reporting of leisure-time sedentary behaviors from the modified 3-day SAPAC among adolescent girls and boys. Other studies have been published on African American, preadolescent girls [17,18] examining correlations between minutes of sedentary behaviors from a modified SAPAC (renamed the GEMS Activity Questionnaire) and mean total minutes of *activity *from accelerometry. The first study (N = 68; age 8-9 years) found no significant correlations between self-reported TV watching and accelerometry, or between other sedentary behaviors minus TV watching and accelerometry [17]. In contrast, the second study of a larger sample of slightly older preadolescent African American girls (N = 172; age 8-10 years) found a significant negative correlation between TV watching and the three-day mean accelerometry minutes of activity (r = -0.19; p = 0.02) [18]. Neither of these studies validated the reported sedentary behaviors against sedentary minutes measured by accelerometry, but rather did comparisons with active minutes.

Cradock et al. (2004) did compare minutes of sedentary behavior by self-report to that of accelerometry [15]. In a study of 54 middle school students (age 13.8 ± 0.7 years) they found a significant correlation between the proportions of time spent in sedentary behaviors (< 1.5 METs) from an interviewer-administered 24-hr recall and TriTrac accelerometry (r = 0.48; p < 0.05). There were many differences between that study and the one reported here; however, likely explanations of the higher correlation found by Cradock et al. (2004) are the use of a different self-report instrument and the fact that the recall was interviewer-assisted rather than self-administered.

In a more recent study of 447 Boy Scouts (age 10 to 14 years), there was no statistically significant correlation between the 3-day average minutes of sedentary behavior from accelerometry and the self-reported sedentary behavior during the previous day and usual sedentary behavior (r = 0.063 and r = 0.094, respectively) from a modified version of the SAPAC [7]. However, further regression analyses found an inverse association between social desirability and self-reported sedentary behavior from the previous day (β = -0.15, P = 0.008).

Findings in the present study suggest the three-day SAPAC did not sufficiently capture sedentary behaviors in adolescent girls and boys, with mean levels generally underestimated compared to accelerometry. The use of only four sedentary behaviors from the modified SAPAC may have contributed to the underestimation of sedentary pursuits measured by accelerometry. However, studies in adolescents and adults [7-9,19] have also shown an underestimation of the self-reported minutes of sedentary behaviors. Sedentary behaviors may be more difficult to remember than activities of higher intensity [9]. Compared to adults, adolescents may have more difficulty recalling and processing intermittent complex information about past sedentary behavior [5,20]. In addition recall bias, social desirability has been associated with underreporting of sedentary behaviors in adolescents boys [7].

Bias was observed in the hypothesized direction in self-reported sedentary behavior associated with body weight status, although the bias was statistically significant only in overweight girls and normal weight boys. Previous reports have shown that it is important to consider recall and reporting bias when assessing behaviors in children and adolescents [1]. Social pressure may influence overweight adolescent girls to underreport sedentary behavior to a greater extent than other groups [21]. However, the effects of social desirability on reports of sedentary behavior by weight status have not been evaluated.

The current study benefited from multiple days of sedentary behavior recall and objective measurements, which allowed for a more accurate assessment of usual sedentary behavior. The diversity of the sample studied is also a strength of the study. One weakness of this study is that sedentary behavior was not assessed during school. Had sedentary minutes during school also been reported it is possible that correlations would have been higher. However, this does not alter the poor performance of the questionnaire for measuring minutes of sedentary behavior outside of school.

Moreover, to our knowledge this is the first study to use Bland-Altman plots with three different analytical strategies to evaluate the comparability between the two measures of sedentary behavior. The agreement between the self-report and accelerometer appeared to be more precise using the residuals method (Figure 1c.). This plot showed less dispersion (within ± 1 SD of the mean difference) in the estimates of sedentary behavior between self-report and accelerometry.

Several investigators have used SAPAC to assess sedentary pursuits in adolescents [12-14]. Our results indicate that such studies should be interpreted with caution since the validity of the SAPAC to assess sedentary behavior appears to be invalid. The findings of the current study points to the likelihood of misclassification of sedentary behavior by self-report among adolescents. The implications of misclassification of sedentary behaviors are twofold. First, using a modified version of the SAPAC to capture sedentary behaviors would likely lead to an underestimation of the prevalence of inactivity among adolescents. Secondly, the association between self-reported sedentary behaviors and outcomes of interests such as excess body weight would be attenuated. Both of the implications have the potential to delay action of interventionists and policy makers. For example, interventionists and policy makers may not recognize the magnitude of the problem of sedentary behavior in youth and fail to develop programs or institute policies designed to reduce this behavior. These findings highlight the need for further development of methods for assessing sedentary behaviors which might include questionnaires that query more sedentary pursuits and a format that combines a checklist with time-cues for better recall such as start and stop times for common TV shows. The current availability of accelerometry as a criterion measure with which to compare self-report instruments to assess sedentary behavior should lead to the development of better tools.

In conclusion, large epidemiological studies require physical activity assessment tools that have both low-cost and low subject burden. Therefore, self-report instruments remain the most often used technique to assess physical activity in large samples. However, results from self-report instruments are so poor that conclusions reached in these studies come into question. It is recommended that accelerometers be used whenever possible, or, at a minimum, in a subset of the target population of the study to create prediction equations for self-reported sedentary behavior assessments. The contributions of this research may lead to better methods for measuring self-reported sedentary behavior to support this important area of public health research.

## Research Methods and Procedures

### Participants

This study was conducted as part of the feasibility phase of the Trial of Activity for Adolescent Girls (TAAG), a randomized controlled trial designed to "determine if an intervention that provides opportunities for physical activity linking schools to community organizations can reduce the age-related decline in moderate to vigorous physical activity (MVPA) in middle school girls" [22]. In Spring 2002, a convenience sample of 224 boys and girls enrolled in 6th through 8th grades were recruited from six field centers in diverse locations across the United States: Arizona, California, Louisiana, Maryland, Minnesota, and South Carolina. Each center recruited a convenience sample of 30 girls and 14 boys from diverse ethnic groups and activity levels. Care was taken to recruit at least 10 girls involved in organized sports and physical activities from each field center to insure a broad range of activity levels which was important for the primary outcome variable (MVPA) of the substudy.

Of the 224 students recruited, five were excluded due to missing questionnaire data, 11 were excluded due to missing accelerometer data, and 16 were excluded because they did not meet the study adherence criteria for the number of hours per day the accelerometer was worn (minimum of 11.2 hours on weekdays and 7.2 hours on weekend days). Two additional students were excluded for missing demographic data. The final analysis sample included 190 participants (122 girls and 68 boys; 84.8% of students recruited).

This study was approved by the Institutional Review Boards at each field center. In addition, approval was obtained from the school or school district. Informed consent was obtained from a parent or guardian and informed assent was obtained from each participant. The University of North Carolina at Chapel Hill was the study coordinating center.

### Data collection schedule

All participants were fitted with accelerometers to collect 3 days of objective data for comparison with the self-report data. Each participant used a modified SAPAC to recall sedentary behaviors for each of the previous 3 days. One hundred and forty students (97 girls and 43 boys) were randomly assigned to complete the modified SAPAC on Tuesday to recall their behaviors on Saturday, Sunday and Monday, while 84 students (48 girls and 36 boys) completed the questionnaire on Wednesday for Sunday, Monday, and Tuesday. This uneven distribution across days was due to collection of data on an alternative questionnaire, which was not part of this investigation. Height, weight and demographic information were collected on study day 1.

### Demographic and anthropometric variables

A questionnaire was used to assess age and ethnicity. The students had the option of selecting one or more ethnic categories or selecting 'other' and specifying ethnicity. Height was measured to the nearest 0.1 cm using a portable stadiometer (Shorr Height Measuring Board, Olney, MD). Weight was measured to the nearest 0.1 kg on an electronic scale (Seca, Model 770, Hamburg, Germany). Weight status groups were determined using the 2000 Centers for Disease Control and Prevention growth charts for children and adolescents [23]. Normal weight was defined as BMI percentile for age and sex < 85^th ^percentile while at risk for overweight plus overweight (hereafter referred to as "overweight") was defined as BMI percentile for age and sex ≥85^th ^percentile [24].

### Self-reported sedentary behavior

A modified 3-day SAPAC was administered to groups of students in a classroom setting, and detailed instructions were given to provide contextual cues to enhance recall. Specifically, the students were asked to think about their activities for each day prior to recording their responses. The original SAPAC [11], for which validity was established for the physical activity portion of the instrument compared to accelerometry (r = 0.33, p < 0.001), assessed two categories of sedentary activities: 1) TV/video and 2) video games and computer games and was designed for one day of activity recall. Based on information obtained during the TAAG feasibility period about common sedentary behaviors among adolescents, two additional questions were added to the activity-based questionnaire for this study: 1) computer/internet use and 2) talking on the phone. Students recorded the number of hours and minutes spent in the four types of sedentary behaviors.

Sedentary behavior was assessed only during hours in which the students were not in school. On weekends, time spent in the four sedentary behaviors was reported for morning, between lunch and dinner, and after dinner. The maximum number of sedentary minutes that could be accrued on weekend days was set at 300 minutes for the morning interval, 300 minutes for the interval between lunch and dinner, and 420 minutes for the after-dinner interval. These intervals were arbitrarily set defining 7 am to 12 noon as morning, 12 noon to 6 pm as the interval between lunch and dinner, and 6 pm to midnight as the after dinner interval. On weekdays, time spent in sedentary behaviors was ascertained before school and after school. On weekdays, the maximum number of sedentary minutes that could be accrued was set at 120 minutes before school (range: 0-120 minutes) and 540 minutes after school (range: 0-540 minutes). These maxima were set using the approximate start and end time for school days as indicated by the average school bell schedule. Thus, the maximum amount of sedentary time that could be accrued was 660 minutes for weekdays and 1020 minutes for weekend days.

### Criterion measure of sedentary behavior

The criterion measure of time spent at the sedentary level was assessed using the Actigraph^® ^accelerometer, formerly the CSA accelerometer (Model 7164, Manufacturing Technology Inc. [MTI], Ft. Walton Beach, FL). The Actigraph accelerometer has been calibrated for use as an objective measure of sedentary behavior in children and adolescents [2,3]. Data were collected as the average number of counts in 30-second epochs, and bounds for sedentary behavior were set using results from Treuth et al (2004) [25]. In that study seventy-four 8^th ^grade girls performed activities of various intensity levels while wearing an Actigraph and a portable indirect calorimeter. The upper bound for low intensity (sedentary) activity was found to be 50 counts · 30 sec^-1 ^epoch based upon sensitivity and specificity analyses. We considered sustained (20-minute) periods of zero counts to represent times when the monitor was not being worn and these counts did not contribute to minutes of sedentary behavior, which is standard in the literature [25]. Furthermore, criteria for daily adherence to monitor wear time protocols were established. More specifically, data from monitors with < 7.2 hours on weekend days and < 11.2 hours on weekdays were deleted from the accelerometer data files [25].

### Statistical Analyses

Time-matched intervals from the self-report and the accelerometer for sedentary behaviors were used to compare the two instruments. For example, on weekend days the morning interval of 7 am to 12 noon was time-matched with the minute-by-minute accelerometer data that corresponded with the same time period. The sedentary behavior values (minutes) were summed for each day and averaged across all 3 days. Analyses were stratified by sex and weight status. T-tests were used to evaluate differences in means. Spearman rank-order correlations and Pearson product-moment correlations were used to compare minutes of sedentary behaviors from the modified SAPAC to those measured using accelerometry. Correlations were examined with minutes of sedentary behavior expressed as: 1) crude minutes, 2) percentage of minutes measured spent at the sedentary level, and 3) sedentary minutes adjusted for total minutes measured using the residuals method [16]. The latter method uses the residuals from models regressing total minutes measured on sedentary minutes. A residual value is calculated for each participant and the sample mean number of sedentary minutes is added to that value. Overall correlations were calculated using the three-day weighted average of the Fisher's Z transformation of each day's correlation [26]. This procedure allows for the deattenuation of the correlation due to correlated error between the estimates. Bland-Altman plots were used to examine the difference or bias between self-reported and accelerometry-measured sedentary behavior [27]. For comparison of the three analytical strategies, the Bland-Altman plots were standardized to one standard deviation from the mean difference between self-report and accelerometer. Although Bland-Altman plots are a commonly used statistical method used in the field of physical activity research, there is controversy around its ability to accurately assess bias between two instruments [28]. Therefore, regression analyses were also performed to assess bias. Repeated measure ANOVAs that accounted for site and school clusters of students were performed using SAS PROC MIXED [29]. To examine the relationship between self-reported sedentary behavior and accelerometer-measured sedentary behavior, self-reported sedentary behavior as the dependent variable and accelerometer-measured sedentary behavior ad the independent variable were used in the model. Covariates used the in the model included day, age, grade, sex, ethnicity, and weight status. All analyses were performed using SAS Version 8.2 [30].

## Competing interests

The authors declare that they have no competing interests.

## Authors' contributions

OA contributed to the design of the study, the statistical analysis, the interpretation of the data, and the drafting of the manuscript. JS, RM, DW, LL, MS, DY contributed to the data interpretation and revision of the manuscript. DC contributed to the statistical analysis and interpretation of the data. All authors have read and approved the final manuscript.
